# OSA: Treatments beyond CPAP

**DOI:** 10.3390/jcm11195938

**Published:** 2022-10-08

**Authors:** Giovanni Cammaroto, Andrea Migliorelli, Claudio Vicini

**Affiliations:** 1 ENT Unit, Morgagni Pierantoni Hospital, 47121 Forli, Italy; 2 ENT Unit, University of Ferrara, 44121 Ferrara, Italy

Obstructive Sleep Apnea (OSA) is a serious and underestimated respiratory sleep disorder that affects approximately 24% of men and 9% of women, and over a billion people worldwide [1,2].

OSA is characterized by repeated complete (apnea) or partial (hypopnea) reduction of airflow in the upper airway during sleep [1,3,4].

This condition has been proved to be an independent risk factor for hypertension, myocardial infarction and stroke [1,2,3,5] and has also been reportedly related to type II diabetes, Parkinsonism, Alzheimer’s disease and dementia [3,5].

During the recent pandemic, it has been demonstrated that untreated OSA is a risk factor for COVID-19 disease, which occurs in a more severe pattern and with increased mortality [6].

Furthermore, daytime sleepiness typically complained by OSA patients has a significant impact on their quality of life and increases the risk of motor vehicle accidents [1,2,4].

Diagnosis is traditionally based on medical history, Epworth Sleepiness Scale (ESS) and sleep tests such as polysomnography [2,3,4].

Careful analysis and correction of unhealthy habits (diet, exercise, sleep hygiene, use of alcohol or sedatives) and unfavorable sleep position are generally recommended [2,5].

Continuous Positive Air Pressure (CPAP) is considered the gold standard treatment of this disease, especially in case of moderate to severe OSA [1,2,4,5,7].

Despite the proven effectiveness of CPAP, a high percentage of patients shows low adherence, with reduced therapeutic efficacy as an effect [1,2,4,5,7].

Therefore, numerous efforts have been made to find alternative effective therapies during the last decades.

One of the most common alternative therapies for OSA is Oral Appliance treatment (OA).

Currently, treatment with OA is indicated in the event of CPAP failure or if requested by patients, preferably in non-obese individuals [1,5].

The mandibular advancement device (MAD) is certainly the most frequently adopted and investigated in the literature.

Mandibular advancement results in an overall reduction of upper airway collapsibility due to both an increase in lateral diameter, posterior to the soft palate, and an anterior movement of the tongue [1].

Polysomnographic indexes generally improve with the use of OA, although residual OSA is often registered (Apnea-Hypopnea index (AHI) > 5). On average, a reduction in AHI of about 50% is obtained, with at least one-third of patients achieving a complete response [1].

However, increasing evidence shows that, despite the residual AHI with OA therapy, similar clinical outcomes (hypertension, cardiovascular diseases, neurological diseases) can be achieved when compared to CPAP. This finding could be explained by a higher adherence to OA treatment.

Side effects of OA therapy are usually temporary and include dry mouth, excessive salivation, or soft tissue irritation [1].

To better select patients eligible for this therapy, drug-induced sleep endoscopy (DISE) can be performed. A DISE finding of collapse behind the soft palate and/or a posterior positioned tongue are anatomical predictors of a favorable response. Hypopharyngeal collapse, on the other hand, appears to be a negative prognostic factor [1].

However, OA is still commonly considered a second-line therapy, especially in the case of severe OSA [1,5].

Another treatment option for OSA is surgery, which has attracted great scientific interest in recent years [2,3,4,5,7,8].

Removing obstructive tissue and improving the airway cross-section are among the main targets of sleep surgery [2,3].

Soft tissue surgery for OSA classically involves the treatment of the palate and base of the tongue (BOT) [2,3].

The most widespread palate procedure is probably Uvulopalatopharyngoplasty (UPPP).

UPPP consists of tonsillectomy (if not previously performed) followed by partial resection of the soft palate and uvula [3].

Due to the significant fibrotic and stenotic complications secondary to this operation and the introduction of barbed sutures, barbed repositioning pharyngoplasty (BRP) has become one of the most performed palate procedures nowadays [3].

BRP consists of a tonsillectomy (if not previously performed) followed by a bidirectional suture. A needle is introduced at the level of the posterior nasal spine and then passed laterally underneath the soft palate, and then around the pterygomandibular raphe. The needle is reintroduced submucosally around the pterygomandibular raphe and reaches the tonsillectomy bed. The thread is therefore passed around the upper part of the palatopharyngeal muscle and exits near the posterior pillar mucosa [3]. The procedure is repeated several times in order to anchor the lateral pharyngeal wall to the pterygomandibular raphe. BRP is performed on both sides and does not require knots [3].

This technique involves the use of a barbed suture to avoid the resection of muscular and mucosal structures. The aimed functional outcome is the increase in the transversal and anteroposterior upper airway diameters [3].

BRP seems to allow shorter operating time, greater rigidity of the soft palate, and rare minor complications, such as extruded thread, mild bleeding, suture rupture, and minimal pharyngoplasty dehiscence [3].

Therefore, BRP has proven to be an easy, non-time-consuming, safe, and effective procedure in the treatment of moderate to severe OSA [3].

In the last decades, the use of Trans-Oral Robotic Surgery (TORS) for the treatment of OSA has become increasingly important, with satisfactory results reported also in moderate and severe OSA [2,4,7,8]. TORS is a minimally invasive approach that allows partial resection of the BOT (including lingual tonsils and, eventually, muscular layers) and supraglottoplasty [2,4,7,8].

The main indication for TORS is hypertrophy of the lingual tonsils (Friedman type 3 or 4). This technique requires a minimum distance of 1.5 cm between the upper and lower teeth and an adequate surgical exposure [2].

One of the major complications is hemorrhage, which requires surgical revision in 2.5% of cases [2]. Other complications described in the literature are usually transient and consist of hypogeusia, pharyngeal edema, and pain, usually dealt with by conservative measures [2].

Several advantages related to the adoption of robotic surgery can be mentioned: excellent visualization of the surgical field due to the magnification of the robotic system and the high-definition camera, three-dimensional vision, motion scaling, and tremor filtering technology. In addition, robotic articulated arms allow us to reach more remote areas generally not approachable with conventional techniques of surgery [7,8].

On the other hand, high costs and longer surgical time limit the diffusion of robotic surgical systems [8].

Furthermore, it should be emphasized that upper airway collapse is often multilevel, with retropalatal and retroglossal regions being the most frequently involved sites [3]. Simultaneous obstruction of these two levels was observed in 25–33% of cases [3].

Frequently, treatment failure following single-level surgery is determined by the presence of an untreated secondary site of the collapse.

Therefore, a careful assessment to identify all anatomical sites of airway collapse is mandatory for the successful management of OSA patients [2,7].

This assessment is commonly performed by means of DISE, crucial for optimal surgical planning [2,3,7].

It was recently shown that surgical planning based only on the Muller maneuver changed in 40–50% of patients after DISE [2,3,7]. In this scenario, multilevel surgery appears a viable option and might be performed in one stage [7]. In particular, the combination of TORS and BRP has proven to be largely effective in the treatment of moderate to severe OSA [2,3,4].

Furthermore, evidence shows that multilevel treatment and CPAP have a comparable positive impact on the patient’s quality of life. This finding might be related to the main advantage of surgery over CPAP: adherence is not required [4].

Nowadays, in rare cases of CPAP intolerance and failure after soft tissue surgery, more invasive surgical treatments such as Maxillo-Mandibular Advancement (MMA) are suggested. MMA is a very effective technique for upper airway widening and stiffening. On the other hand, this treatment tends to modify patients’ physiognomy and might lead to some post-operative complications such as facial paresthesia [4].

Finally, treatments targeting the dilator muscles of the upper airways need to be discussed [1,5].

In particular, the genioglossal muscle (GG) plays a very important role in preventing upper airway collapse during sleep [5]. In the transition from wakefulness to sleep, the activity of the GG decreases and this leads to upper airway obstruction in OSA patients [1,5].

Hypoglossal nerve stimulators (HNS) have recently been introduced in the market [2,5]. The hypoglossal nerve innervates all muscles of the tongue except the palatoglossus and its stimulation leads to tongue protrusion and, as consequence, the opening of the upper airways [2,5].

These devices include an implantable pulse generator (IPG), surgically placed in a subcutaneous pocket at the superficial level of the pectoralis major, and an electrode cuff connected to the IPG that surrounds the distal portion of the hypoglossal nerve [5].

In addition, some HNS devices present an implantable chest sensor that monitors respiratory effort [5]. The surgical implantation procedure is performed under general anesthesia [5].

Initial studies have shown an improvement of polysomnographic indexes with improved AHI even in patients with moderate to severe OSA [5]. To date, further studies are needed to better select patients who are potential candidates for this treatment, since more than 30% of patients do not seem to respond properly [5].

Moreover, these devices require invasive treatment, and periodic servicing and are burdened by high costs [2,5]. In addition, this technique cannot be performed in obese patients [2].

The use of myofunctional therapy (MT) has also been suggested to increase the tone of the upper airway musculature. This approach consists of a series of exercises to improve the sensitivity, proprioception, motility, coordination, and strength of the orofacial structures [5].

MT has been shown to reduce AHI and daytime sleepiness symptoms.

MT can be prescribed as a stand-alone therapy in patients with mild OSA or in combination with other treatments in patients with moderate to severe OSA, although further evidence is needed in order to prove its efficacy [5].

Lastly, the first pharmacological approaches to OSA have been described recently. Unfortunately, only the therapeutic combination with a norepinephrine reuptake inhibitor and a muscarinic blocker has shown promising outcomes. These drugs act as GG tone modulators with subsequent reduction of AHI and increased nocturnal oxygenation [5].

However, further studies are needed to demonstrate the effect of the drugs over time and to identify which category of subjects might respond more consistently [5].

Numerous effective and exciting alternative treatment options to CPAP have been developed in recent years.

The series of articles that have been analyzed in this editorial highlights the growing interest in literature, although the main limitation still appears the lack of accurate outcome predictability.

A better interpretation of the pathophysiological mechanisms of OSA and a deeper understanding of the phenotypic characteristics of individuals affected by this disease are still highly needed in order to tailor an individualized and effective treatment.
